# Unmet needs in the international neuroendocrine tumor (NET) community: Assessment of major gaps from the perspective of patients, patient advocates and NET health care professionals

**DOI:** 10.1002/ijc.32678

**Published:** 2019-10-25

**Authors:** Simone Leyden, Teodora Kolarova, Catherine Bouvier, Martyn Caplin, Siobhan Conroy, Phillipa Davies, Sugandha Dureja, Massimo Falconi, Piero Ferolla, George Fisher, Grace Goldstein, Rodney J. Hicks, Ben Lawrence, Yoshiyuki Majima, David C. Metz, Dermot O'Toole, Philippe Ruszniewski, Bertram Wiedenmann, Ronald Hollander, International Neuroendocrine Cancer Alliance (INCA)

**Affiliations:** ^1^ The Unicorn Foundation Mosman Australia; ^2^ International Neuroendocrine Cancer Alliance (INCA) Boston MA; ^3^ NET Patient Foundation Leamington Spa United Kingdom; ^4^ Neuroendocrine Tumour Unit Royal Free Hospital London United Kingdom; ^5^ Unicorn Foundation Auckland New Zealand; ^6^ Royal Free Hospital London United Kingdom; ^7^ Department of Nuclear Medicine & Molecular Imaging Fortis Memorial Research Institute Gurgaon Haryana India; ^8^ Pancreatic Surgery Unit Pancreas Translational & Clinical Research Centre, San Raffaele Scientific Institute ‐ “Vita e Salute” University Milan Italy; ^9^ Department of Internal Medicine and Endocrine Sciences University of Perugia Perugia Italy; ^10^ Stanford University School of Medicine Stanford CA; ^11^ The Carcinoid Cancer Foundation White Plains NY; ^12^ The Sir Peter MacCallum Department of Oncology University of Melbourne Parkville VIC Australia; ^13^ Discipline of Oncology Faculty of Medical and Health Sciences, University of Auckland Auckland New Zealand; ^14^ Pancreatic Cancer Action Network Tokyo Japan; ^15^ Division of Gastroenterology Perelman School of Medicine, University of Pennsylvania Philadelphia PA; ^16^ National Centre for Neuroendocrine Tumours St. Vincent's University and Department of Clinical Medicine, St. James Hospital and Trinity College Dublin Ireland; ^17^ Division of Gastroenterology and Pancreatology Beaujon Hospital Paris France; ^18^ Department of Hepatology Gastroenterology and Endocrinology, Charité Medical School Berlin Germany; ^19^ Neuroendocrine Tumor Research Foundation (NETRF) Boston MA

**Keywords:** neuroendocrine tumor, perspective, standards of care, survey, unmet needs

## Abstract

Due to the increasing incidence and prevalence of neuroendocrine tumors (NETs), there is a need to assess any gaps in awareness and care. A survey was undertaken in 2017 to identify perceived unmet needs from the perspectives of patients/families, patient advocates and health care professionals (HCPs). The survey consisted of 33–37 questions (depending on type of respondent) across four areas: information, care, treatments and research. In total, 443 participants from 26 countries responded: 338 patients/families, 35 advocates and 70 HCPs. Perceived unmet needs regarding provision of information at diagnosis differed between groups. While 59% of HCPs believed they provided sufficient information, informational needs were mostly/fully met for only 30% of patients and 18% of advocates. Additionally, 91% of patients and 97% of advocates felt that patients had to search for information themselves. Availability of Gallium‐68‐Dotatate PET/CT scan was limited for the majority of patients (patients: 73%; advocates: 85%; HCP: 86%), as was access to treatments, particularly peptide receptor radionuclide therapy (patients: 42%; advocates: 95%; HCPs: 77%). All groups felt that standards of care, including psychological needs and diagnosis of mental health, were not fully met. Although about two‐thirds of patients were managed by a multidisciplinary team, 14% of patients reportedly did not have enough contact. All groups supported more patient involvement in research; patients and advocates prioritized improvement in diagnosis and HCPs focused on clinical trials. This survey revealed significant unmet needs but differing perceptions regarding these among the groups. There is a need for investigation and collaboration to improve standards of care for NET patients.

## Abstract 2

**What's new?**

Even though the incidence of neuroendocrine tumors (NETs) has been rising worldwide, the current management of patients varies considerably, potentially leaving many with suboptimal care. An international survey was carried out in 2017 to investigate unmet needs in the NET patient community. The survey revealed that patients perceive numerous unmet needs in key areas including provision of information, diagnostics and treatment access, care standards, and research involvement. While healthcare professionals were aware of these gaps, they generally underestimated their magnitude. Patients and healthcare professionals need to work together to improve the lives and prospects of the increasing numbers of patients.

## Introduction

Neuroendocrine tumors (NETs) are neoplasms arising from cells of the neuroendocrine system occurring throughout the body.^1^, ^2^ Around 20% of NETs are associated with inherited genetic syndromes such as multiple endocrine neoplasias (MEN1/MEN2), Von Hippel–Lindau (VHL) and neurofibromatosis (NF1).^3^ Pheochromocytomas and paragangliomas are also rare but unique NETs.^4^ Although the incidence of NETs varies from 1.51 to 6.98 per 100,000 annually, studies show an increasing trend in the incidence worldwide,^2^, ^5^, ^6^ possibly due to earlier and improved diagnosis. Many patients, however, still experience diagnostic delays^5^, ^7^ and are often misdiagnosed due to nonspecific symptoms.^8^, ^9^ Indeed, the first Global.NET Survey found almost 60% of NETs are advanced when diagnosed.^7^ Consequently, survival rates can vary widely, ranging from 6 months to >30 years.^2^, ^6^, ^10^ If symptoms are not managed effectively, patients may be frequently hospitalized^6^ and experience reductions in quality of life (QoL).^11^

Studies have highlighted a number of unmet needs and ongoing challenges.^12^ The current management of patients with NETs varies considerably, potentially leaving many with suboptimal care. Compared to other cancer types, the patient experience for NET patients is markedly different.^13^ Progress is hindered by limited understanding of NET pathogenesis, lack of animal models, targets for therapies and prognostic factors, evolving changes in classification and treatment staging and limited investment in research.^6^ Finding the right sequence of treatment can also be difficult due to the variability of individual patient disease progression and the lack of studies demonstrating optimal sequencing.^6^

The International Neuroendocrine Cancer Alliance (INCA) is a global alliance consisting of 26 patient advocacy and research groups from 22 countries, which supports NET patients and their families. To enable effective collaboration to improve patient access to information, quality care and research, INCA undertook an international survey to identify perceived unmet needs in the management of NETs from the perspectives of three different groups: patients (and their families), patient advocates and health care professionals (HCPs).

## Patients and Methods

Patients (including their families), patient advocates and HCPs completed an online survey between February and March 2017. The survey was comprised of 37 questions for patients, 35 for advocates and 33 for HCPs across four areas—provision of information at diagnosis, standards of care, access to diagnostics and treatment and research—and was tailored toward each group, with some questions differing (Supplementary Appendix S1). Most questions were designed to be cross‐comparable. Participants could skip questions; therefore, answers were based on the total number of respondents for each question. Data from the first Global.NET Patient Survey in 2014 were used to support the identification of unmet needs explored by the survey.^7^ The survey was created with SurveyMonkey® and disseminated within the international NET community by INCA member organizations and advocates. The survey was freely available and not restricted to participants associated with NET Centers of Excellence. Patients could be from the same center as HCPs, but were not matched with their treating HCPs. The survey was conducted in English and all responses were anonymous. As this was an anonymous survey, no ethical approval was required.

## Results

### Participant characteristics

In total, 443 respondents from 26 countries responded: 338 patients/families, 35 advocates (69% NET patients themselves) and 70 HCPs (Supplementary Table S1). HCPs were most likely to work in oncology (39%) or gastroenterology (29%). Nurses accounted for 10% of HCP respondents. Patients and advocates generally had similar disease characteristics (Table 1). Patients answered, on average, 30 of 37 questions, advocates 32 of 35 and HCPs 31 of 33.

**Table 1:** Patient and advocate characteristics

	Patient/family (*n* = 338)	Advocate (*n* = 35)
Patient	88% (296/336)	69% (24/35)
Current age		
<18 years	1% (3/334)	0% (0/35)
18–49 years	30% (100/334)	37% (13/35)
>49 years	69% (231/334)	63% (22/35)
Age at diagnosis		
<18 years	4% (12/336)	0% (0/24)
>49 years	56% (187/336)	29% (7/24)
Type of tumor		
Pancreatic	28% (91/329)	33% (8/24)
Small intestine	23% (76/329)	38% (9/24)
Multiple endocrine neoplasia	17% (56/329)	13% (3/24)
Lung	10% (34/329)	8% (2/24)
Other	22% (72/329)	8% (2/24)

### Informational needs at diagnosis

**Figure 1:**
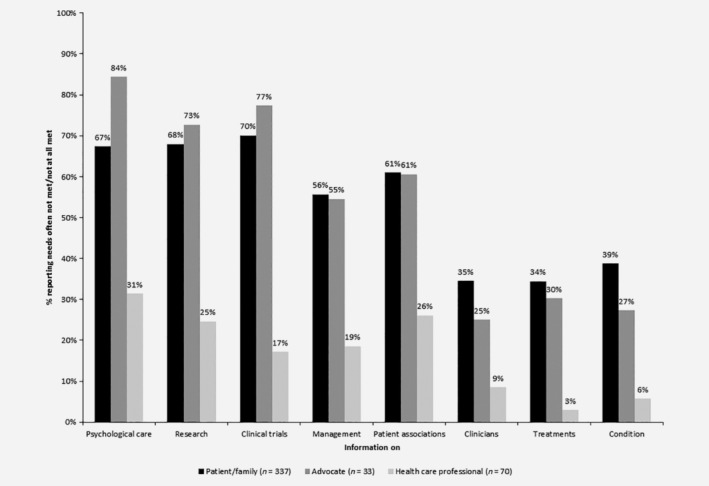
Unmet informational needs at diagnosis. Spearman correlation (in terms of ranked responses across questions) between patients/family and advocates was highly significant (*p* = 0.007), whereas between patients/family and HCPs was not significant (*p* = 0.071). The correlation between advocates and HCPs was significant at the 5% level (*p* = 0.037). Formal statistical analysis of individual questions was not undertaken due to the imbalance in respondent numbers within each group.

Perceived unmet needs about information at diagnosis differed between groups (Fig. 1). While HCPs felt they provided patients with sufficient information (59%), informational needs were mostly or fully met for only 30% of patients and 18% of advocates. In addition, 84% of HCPs felt able to give patients sufficient information on NETs and 67% felt patient needs regarding the amount of discussion time were mostly or fully met. While HCPs were the main source of information for patients (68%), followed by patient organizations (35%), the majority (91%) of patients relied on patient association (69%) or HCP websites (48%) to find information, which mostly or fully met their needs (61% and 44%, respectively). Advocates generally responded similarly compared to patients but overestimated the use of HCPs as important information sources (82%) and patients' use of patient association websites (100%). There was a high level of unmet need for information expressed by patients from across all geographical areas (Supplementary Fig. S1).

### Diagnostics and treatment needs

Availability of key diagnostic and therapeutic tools was perceived to be limited by all respondents wherever they were based geographically (Supplementary Tables S2 and S3); particularly Gallium‐68‐Dotatate PET/CT scans and peptide receptor radionuclide therapy (PRRT) was most often cited as unavailable. Surgery was widely available for patients according to advocates (0% reporting as unavailable), but 16% of HCPs and 19% of patients perceived that it was not available.

Patients attributed limited availability of treatments to their health care system (28%), followed by lack of referral (19%), financial reasons (treatment not covered by insurance [17%] or inability to afford treatment [18%]). A higher proportion of advocates attributed the unavailability of treatment on the health care system (67%) and inability to afford treatment (57%). Distance to the treatment center was another common reason reported by advocates (48%). In line with this, 30% of patients had to travel more than 300 km/186 miles for treatment, although, according to advocates (48%) and HCPs (46%), this number may be underestimated. Advocates also perceived a higher need for patients to travel abroad for treatment (patients: 14%; advocates: 79%). Only 10% of HCPs believed that funding affected the availability of appropriate diagnostic testing (needs often not met/not at all met). Funding, however, was highlighted as a limiting factor for participation in research (21%) and clinical trials (21%), as well as teaching time and training (21%).

### Care needs

**Figure 2:**
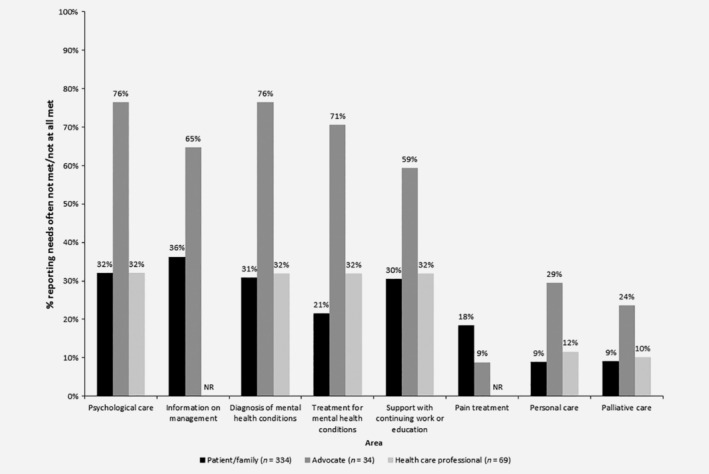
Unmet care needs as perceived by patients and advocates. NR, not reported. The total number of respondents in each participant group differed slightly for each question because participants were allowed to skip questions. Percentages shown are calculated based on actual numbers. Health care professionals were not asked about “information on management” and “pain treatment.” Spearman correlation (in terms of ranked responses across questions) was significant at the 5% level between patients/family and HCPs (*p* = 0.021) and advocates and HCPs (*p* = 0.016), and was borderline significant between patients/family and advocates (*p* = 0.050). Formal statistical analysis of individual questions was not undertaken due to the imbalance in respondent numbers within each group.

Approximately, a quarter of patients and HCPs (23% and 25%, respectively) and half (51%) of advocates believed that patients' care needs were often not fully met (Fig. 2). A higher proportion of advocates (35%) believed that patients were not given all the necessary information about their condition and treatment options compared to patients (20%). Advocates also perceived that more patients had difficulty finding this information (16%). Moreover, 76% of advocates believed patients' psychological care needs were either often not met (38%), or not at all met (38%), while 71% felt their treatment needs for diagnosed mental health conditions were often (44%) or not met at all (27%). Interestingly however, patients and HCPs perceived mental health needs to be better addressed, with only 32% in each group of the opinion that psychological care needs were often not met or not met at all. Since patients most often contact patient organizations when in distress, this might explain the differing viewpoints of advocates *versus* patients and HCPs. Despite reportedly needing a non‐HCP home caregiver, 50% of patients reported that they lacked access to one. Additionally, 29% believed that caregivers did not fully meet the needs of patients, even when a professional caregiver was used (33%).

Two‐thirds of patients (66%) reported that a multidisciplinary team (MDT) was available to them (advocate: 94%; HCP: 70%). All groups, however, felt that sufficient contact with the MDT (≥1 per month) was an issue. When asked whether patients feel like they are truly a partner alongside HCPs in treatment and care decisions, 41% of advocates and 23% of patients felt that needs were not fully met compared to 18% of HCPs. Access to an MDT was reported by 80% of respondents in Europe, 72% in Australia/New Zealand, 47% in North America and 52% from other represented countries (see Supplementary Table S1 for full list of included countries).

### Research needs

A greater number of patients and advocates (68% and 62%, respectively) believed that patient involvement in research design is important compared to HCPs (46%). All groups felt that patients were not sufficiently involved (patients: 53%; advocates: 82%; HCPs: 57%). The most important unmet needs for NET research were: putting NETs on equal footing with the research of other major cancers (patients: 74%; advocates: 74%; HCPs: 49%), clinical trials to improve current treatments/test new ones (patients: 74%; advocates: 74%; HCPs: 36%) and making sure published results of a research initiative are understood by patients (patients: 65%; advocates: 68%; HCPs 26%). Overall, 34% of HCPs felt it was important to include patients in decisions about the overall strategy and direction of research funding. Research into improving diagnosis was ranked as the top priority for patients and advocates; whereas HCPs prioritized clinical trials to improve current treatments/test new ones. All groups attached a high degree of importance to research on improving QoL and controlling symptoms.

Overall, 16% of patients reported having participated in a clinical trial, mostly limited to a single trial (76%). In line with this, 55% of advocates reported that health care systems do not sufficiently facilitate enrollment in trials (needs often not met/not at all met). Conversely, the majority of HCPs (79%) reported that their hospitals participated in NET trials, often with ≥3 ongoing (46%) or international (49%) trials. Overall, patients (60%) and advocates (77%) believed that a patient's enrollment in a clinical trial made a positive contribution to treatment regimes. Patients found out about relevant clinical trials largely through patient organizations (90%) and HCPs (84%). However, patients felt these services did not fully meet their information needs regarding relevant clinical trials (14% and 40%, respectively). In total, 41% of HCPs agreed that patients' information needs on clinical trials were often not met/not at all met.

## Discussion

The findings of this survey provide a more detailed picture of the unmet needs of NET patients than identified previously in the literature and highlights key areas for improvement. To our knowledge, this is the first international study that has compared perceptions of patients' unmet needs from the differing perspectives of patients/families, advocates and HCPs. Several important unmet needs were identified. In each of the areas surveyed, less than a quarter of patients felt their needs for more information at diagnosis were fully met. While HCPs generally agreed that there is room for improvement, overall they were considerably more confident about their ability to meet patients' needs than reflected by the experiences of patients themselves.

To date, there are limited data on patients' unmet needs regarding the provision of NET information at diagnosis. A survey of 758 patients in the United States reported that 57% found answers to their NET‐related questions, yet only 45% were given sufficient information after diagnosis and 58% would have liked better direction in obtaining information.^14^ Other surveys also found that patients struggle to source relevant information.^15^, ^16^ This finding is reflected in our survey where many patients reported using the internet and patient associations to obtain information on NETs. A potentially simple and relatively economic strategy would be for HCPs to provide a consistent set of validated, written materials to all NET patients at consultation and signpost patients to local organizations who can provide additional information and support. This would ensure accuracy of information, while also addressing the time constraints faced by physicians' in providing adequate information during limited appointments. However, it must be acknowledged that a proportion of patients will inevitably seek additional information from websites or associations to satisfy their need of a global picture about the disease and treatment process, irrespective of the amount of information provided by a HCP. To ensure that valid, reliable information is provided to patients, all parties need to collaborate to “close the information gap” and develop a more effective approach. One of INCA's major projects is the development of a Global NET Patient Information Pack, which will be available in multiple languages and provide fact sheets covering signs/symptoms, tests, treatments and supportive care to address the wraparound care needs of NET patients. INCA also strongly believes that public funding should be provided to the advocacy community to support their vital work in bridging the gap in provision of information.

Equitable access to innovative technologies and medicines in NETs is a major global challenge.^7^, ^17^ A need for better access to key diagnostic imaging and treatments, including PRRT, was demonstrated in our survey, from the perspective of all three groups. Research shows that improved survival is achieved at centers that adopt a multidisciplinary approach. The median survival of patients within NET Centers of Excellence appears to be over three times longer than in other institutions.^18^, ^19^, ^20^, ^21^ However, as there are likely to be few of these centers, patients may have to face longer travel times. Research and design of shared‐care plans with community‐based oncologists are under development and could provide a template for reducing this burden (CommNETs project). INCA is also in the process of launching a worldwide assessment, “Survey of Challenges in Access to Diagnostics and Treatment for NET Patients” (SCAN), to measure access to diagnostics and treatments and the corresponding financial burden for NET patients from both NET patients and HCP perspectives.

A clear pathway should be established for NETs, directed and supported by national health systems, so that all patients can reliably access a consistent standard of care. Health care systems should also aim to create more specialist centers with a focus on improving NET patient outcomes. Progress has already been made, particularly in Western Europe where ENETS certifies NET Centers of Excellence. Centers of Excellence have also recently been accredited by ENETS in Australia (The Peter MacCallum Cancer Centre), the United States (The University of Iowa) and Israel (Hadassah‐Hebrew University). In countries with formal Health Technology Assessment processes, patient representatives should be routinely consulted as part of negotiations. Patient association campaigns have been critical toward improving access to PRRT in several countries including Japan, Australia and New Zealand. INCA believes that global cooperation between NET patient associations and medical communities may achieve a greater influence, especially in the appraisal of new technologies.

Research suggests that delivery of consistent and appropriate standards of care in NETs may be suboptimal worldwide,^7^, ^17^ which is consistent with the findings of our survey. Our results also highlight that supportive care, for example, for mental and emotional health as well as nutritional needs, is lacking and that contact needs with an MDT are unmet for about 15 in 100 patients. Improved utilization of existing resources by adopting a patient inclusive MDT, as highlighted in the first Global.NET Patient Survey, could help address these issues. Patients with access to an MDT reported numerous improvements, including patient satisfaction with care, relationships with HCPs and knowledge about their condition.^22^

There is an urgent need to increase awareness and specialized education in NETs among all relevant HCPs, as this may facilitate faster recognition, diagnosis and referral for patients. NET MDTs can also benefit from inclusion of a NETs specialist nurse who can dedicate more time to patient education and help to provide holistic support. Surveys have found that the presence of a specialist nurse has the potential to support a more positive patient experience.^23^, ^24^, ^25^ Specifically, patients have highlighted the importance of specialist nurses for psychological care, which is a key unmet need.^13^ Yet, research suggests that many nurses do not have the confidence to help and support NET patients.^26^ Some patient organizations address this by making nurse resources available *via* telephone and by raising money to support training and specialist nurse posts. Wherever possible, NET nurses should be encouraged through the medical education system. Support for the MDT model must be included in payment and insurance systems to ensure they can be adequately funded and maintained.

Patient involvement in research was seen as important by all groups, although the focus of research differed between patients and HCPs. Historically, NETs have not been the focus of rigorous clinical research owing to their perceived rarity; however, with increasing incidence worldwide, it is imperative research surrounding NETs parallels that of other major cancers.^2^, ^5^ HCPs need to raise awareness about clinical trials in the patient community, thereby increasing enrollment and ensuring that patients are kept up‐to‐date. Collaboration between HCP and advocates is crucial to encourage more patient involvement.

Our study and data from the U.K. NET Patient Experience Survey^13^ indicate that the unmet needs of NET patients might be substantially greater in comparison to the situation in more common cancer types. The U.K. NET Patient Experience Survey reported that NET patients were less likely to be given enough information about their condition and treatment compared to other cancer patients (71% *vs*. 88%).^13^ A U.K. study of men with prostate cancer reported only 3.2% felt they had a strong need for more information on their care, diagnosis or treatment, while 48.4% felt this need was met fully.^27^ Similarly, a Japanese study of breast cancer survivors demonstrated that 78.8% were satisfied with information they received on the disease and therapy at diagnosis.^28^ In contrast, in our study only 30% of NET patients reported that their informational needs were mostly or fully met at diagnosis. Other studies have also reported that for patients with more prevalent cancers, satisfaction and accessibility relating to hospitals and both diagnostic and therapeutic tools is superior and travel time shorter than for those with rarer cancers.^27^, ^29^

Our survey has several limitations, which are partly inherent to its design, including provision of the questionnaires in English only. As participants could skip questions, some important answers may have been missed. Furthermore, groups were imbalanced in terms of numbers. Due to the smaller number of advocates, the responses from this group are more likely to be biased. Respondents largely consisted of patients and HCPs connected to NET organizations. Accordingly, their responses may reflect a more “informed” view, and thus, the results may underestimate the actual unmet needs in NETs. Nevertheless, they give important insights into the current delivery of care worldwide. Future surveys might benefit from collecting more information on socioeconomic factors, such as the educational level of patients, and by providing more detailed analyses of geographical differences (by country or region) and stratifying results by NET Centers of Excellence.

In summary, this survey highlights several unmet needs, which appear to be underestimated by HCPs. NET management varies considerably and needs improvement, especially as our results likely underestimate the true gaps due to participant selection. Further research and collaboration is urgently needed to improve earlier diagnosis and expand treatment options. Patients, advocates and HCPs need to work together to improve the lives and prospects of the increasing numbers of patients diagnosed with NETs. Engagement with government agencies to support this patient population should be a priority of advocacy groups and HCPs.

The authors identified the following action points which should be addressed urgently:

- Improve utilization of existing resources, such as including patients in MDTs and providing written information to patients at time of consultation.
- Educate HCPs to enable them to provide patients with sufficient information at diagnosis and about treatment options.
- Provide information and resources to keep patient organization websites up‐to‐date as these are an important source of information.
- Facilitate access to main diagnostic tools and treatments such as Gallium‐68‐Dotatate PET/CT scan and PRRT.
- Provide financial help for patients in need and improve access to caregivers.
- Improve access to MDTs.
- Encourage nurses to specialize in NETs, and ensure training involves teaching around psychological care.
- Increase patient involvement in research by providing information about ongoing trials and facilitate access.
- Conduct further research into NETs, particularly to put NET research on equal footing with other major cancers, ensure earlier diagnosis and to improve current treatments/test new ones.

## Disclaimer

This research was made possible by a grant from the Neuroendocrine Tumor Research Foundation, a non‐profit, charitable foundation dedicated to NET research and support of NET patients.

## List of where and when the study has been presented in part elsewhere

Key data presented as a poster at The European Society for Medical Oncology (ESMO) 2018 Congress, October 19–23, 2018, Munich, Germany; The Asia Pacific NeuroEndocrine Tumour Society (APNETS), November 9–11, 2018, Melbourne, Australia and The European Neuroendocrine Tumor Society (ENETS), March 6–8, 2019, Barcelona, Spain.

## Supporting information


**Appendix S1**: Supporting Information

**Supplementary Figure 1 Patient unmet informational needs at diagnosis by region (online only)**
Aus: Australia; NZ: New Zealand; Europe: Austria, Belgium, Bulgaria, Denmark, Finland, France, Germany, Ireland, Italy, The Netherlands, Norway, Poland, Portugal, Sweden, Switzerland, Spain, United Kingdom; North America: Canada; United States of America; Rest of World: India, Japan, Nepal, Singapore, United Arab Emirates.

**Supplementary Table 1 Geographic breakdown of participants (online only)**
HCP: healthcare professional; UAE: United Arab Emirates; UK: United Kingdom; USA: United States of America.

**Supplementary Table 2 Most common diagnostics and treatments not available (online only)**
CT: computerized tomography; FDG: fluorodeoxyglucose; HCP: healthcare professional; MIGB: meta‐iodobenzylguanidine radiopharmaceutical scan; PET: positron‐emission tomography; PRRT: peptide receptor radionuclide therapy. The total number of respondents in each participant group differed slightly for each question because participants were allowed to skip questions. Percentages shown are calculated based on actual numbers.

**Supplementary Table 3 Most common diagnostics and treatments not available by region (online only)**
CT: computerized tomography; FDG: fluorodeoxyglucose; MIGB: meta‐iodobenzylguanidine radiopharmaceutical scan; PET: positron‐emission tomography; PRRT: peptide receptor radionuclide therapy; Aus: Australia; NZ: New Zealand; Europe: Austria, Belgium, Bulgaria, Denmark, Finland, France, Germany, Ireland, Italy, Norway, Poland, Portugal, Spain, Sweden, Switzerland, The Netherlands, United Kingdom; North America: Canada, United States of America; Rest of World: India, Japan, Nepal, Singapore, the United Arab Emirates. Values shown are calculated based on actual numbers (respondents were allowed to skip questions), and is combined for all participants (patient/advocate/healthcare professional) within each region.

## Data Availability

The data that support the findings of our study are available from the corresponding author upon reasonable request.

## References

[1] Öberg K , Modlin IM , De Herder W , et al. Consensus on biomarkers for neuroendocrine tumour disease. Lancet Oncol 2015;16:E435–46. 26370353 10.1016/S1470-2045(15)00186-2 PMC5023063

[2] Dasari A , Shen C , Halperin D , et al. Trends in the incidence, prevalence, and survival outcomes in patients with neuroendocrine tumors in the United States. JAMA Oncol 2017;3:1335–42. 28448665 10.1001/jamaoncol.2017.0589 PMC5824320

[3] Crona J , Skogseid B . GEP‐NETS UPDATE: genetics of neuroendocrine tumors. Eur J Endocrinol 2016;174:R275–90. 27165966 10.1530/EJE-15-0972

[4] O'Shea T , Druce M . When should genetic testing be performed in patients with neuroendocrine tumours? Rev Endocr Metab Disord 2017;18:499–515. 28965289 10.1007/s11154-017-9430-3 PMC5849652

[5] Hallet J , Law CH , Cukier M , et al. Exploring the rising incidence of neuroendocrine tumors: a population‐based analysis of epidemiology, metastatic presentation, and outcomes. Cancer 2015;121:589–97. 25312765 10.1002/cncr.29099

[6] Tsai HJ , Wu CC , Tsai CR , et al. The epidemiology of neuroendocrine tumors in Taiwan: a nation‐wide cancer registry‐based study. PLoS One 2013;8:e62487. 23614051 10.1371/journal.pone.0062487 PMC3632554

[7] Singh S , Granberg D , Wolin E , et al. Patient‐reported burden of a neuroendocrine tumor (NET) diagnosis: results from the first global survey of patients with NETs. J Glob Oncol 2017;3:43–53. 28717741 10.1200/JGO.2015.002980 PMC5493232

[8] Oronsky B , MA PC , Morgensztern D , et al. Nothing but NET: a review of neuroendocrine tumors and carcinomas. Neoplasia 2017;19:991–1002. 29091800 10.1016/j.neo.2017.09.002 PMC5678742

[9] Santra A , Dutta P , Pothal S , et al. Misdiagnosed case of bronchial carcinoid presenting with refractory dyspnoea and wheeze: a rare case report and review of literature. Malays J Med Sci 2013;20:78–82. 23966830 PMC3743987

[10] Özaslan E , Bayram F , Karaca H , et al. Best prognostic factor of neuroendocrine tumors: grade or stage? A multidisciplinary single‐center study. Turk J Gastroenterol 2016;27:509–14. 27852541 10.5152/tjg.2016.16391

[11] Chau I , Casciano R , Willet J , et al. Quality of life, resource utilisation and health economics assessment in advanced neuroendocrine tumours: a systematic review. Eur J Cancer Care 2013;22:714–25. 10.1111/ecc.12085 PMC4208687 23895457

[12] Modlin IM , Moss SF , Chung DC , et al. Priorities for improving the management of gastroenteropancreatic neuroendocrine tumors. J Natl Cancer Inst 2008;100:1282–9. 18780869 10.1093/jnci/djn275 PMC2538549

[13] NET Patient Foundation : Patient Experience Survey 2015. http://www.netpatientfoundation.org/wp-content/uploads/NET-Patient-Foundation-report-FINAL-1.pdf (Accessed August 2019).

[14] Wolin EM , Leyden J , Goldstein G , et al. Patient‐reported experience of diagnosis, management, and burden of neuroendocrine tumors: results from a large patient survey in the United States. Pancreas 2017;46:639–47. 28328615 10.1097/MPA.0000000000000818 PMC5404397

[15] Devlin L , Jervis N , Bouvier C . Neuroendocrine tumour (NET) patients experiences of support in the community setting across the cancer treatment trajectory. Endocrine Abstracts 2017;52:P10.

[16] Feinberg Y , Law C , Singh S , et al. Patient experiences of having a neuroendocrine tumour: a qualitative study. Eur J Oncol Nurs 2013;17:541–5. 23522828 10.1016/j.ejon.2013.02.003

[17] Öberg K , Castellano D . Current knowledge on diagnosis and staging of neuroendocrine tumors. Cancer Metastasis Rev 2011;30:3–7. 21311954 10.1007/s10555-011-9292-1

[18] Yao JC , Hassan M , Phan A , et al. One hundred years after “carcinoid”: epidemiology of and prognostic factors for neuroendocrine tumors in 35,825 cases in the United States. J Clin Oncol 2008;26:3063–72. 18565894 10.1200/JCO.2007.15.4377

[19] Öberg KE . Gastrointestinal neuroendocrine tumors. Ann Oncol 2010;21:72–80. 10.1093/annonc/mdq290 20943646

[20] Strosberg J , Gardner N , Kvols L . Survival and prognostic factor analysis of 146 metastatic neuroendocrine tumors of the mid‐gut. Neuroendocrinology 2009;89:471–6. 19174605 10.1159/000197899

[21] Singh S , Law C . Multidisciplinary reference centers: the care of neuroendocrine tumors. J Oncol Pract 2010;6:E11–6. 21358944 10.1200/JOP.2010.000098 PMC2988672

[22] Öberg K , Leyden J , Sissons M , et al. Multidisciplinary team in neuroendocrine tumor management: Results from the first global NET patient survey – a collaboration between the International Neuroendocrine Cancer Alliance (INCA) and Novartis Pharmaceuticals. Presented at the European Neuroendocrine Tumor Society (ENETS) 12th Annual Conference, Barcelona, Spain, March 11–13, 2015.

[23] NHS England . National Cancer Patient Experience Survey 2013. https://www.quality-health.co.uk/resources/surveys/national-cancer-experience-survey/2013-national-cancer-patient-exerience-survey/2013-national-cancer-patient-experience-survey-reports/301-2013-national-cancer-patient-experience-survey-programme-national-report/file (Accessed August 2019).

[24] NHS England . National Cancer Patient Experience Survey 2014. https://www.quality-health.co.uk/resources/surveys/national-cancer-experience-survey/2014-national-cancer-patient-experience-survey/2014-national-cancer-patient-experience-survey-national-reports/688-2013-national-cancer-patient-experience-survey-national-report-pdf/file (Accessed August 2019).

[25] NHS England . National Cancer Patient Experience Survey 2017. http://www.ncpes.co.uk/reports/2017-reports/national-reports-2/3579-cpes-2017-national-report/file (Accessed August 2019).

[26] Davies P , Falkerby J , Geilvoet W . Current educational strategies used by nurses caring for NET patients: Electronic survey across 25 countries. Presented at the 15th Annual European Neuroendocrine Tumor Society (ENETS) Conference, Barcelona, Spain, March 7–9, 2018.

[27] Paterson C , Kata SG , Nandwani G , et al. Unmet supportive care needs of men with locally advanced and metastatic prostate cancer on hormonal treatment: a mixed methods study. Cancer Nurs 2017;40:497–507. 28379852 10.1097/NCC.0000000000000482

[28] Miyashita M , Ohno S , Kataoka A , et al. Unmet information needs and quality of life in young breast cancer survivors in Japan. Cancer Nurs 2015;38:E1–E11. 10.1097/NCC.0000000000000201 PMC4612149 25254410

[29] Tanaka H , Ishikawa KB , Katanoda K . Geographic access to cancer treatment in Japan: results from a combined dataset of the patient survey and the survey of medical institutions in 2011. J Epidemiol 2018;28:470–5. 29760321 10.2188/jea.JE20170051 PMC6192973

