# Supplementary material for: Unmet needs in the international neuroendocrine tumor (NET) community: Assessment of major gaps from the perspective of patients, patient advocates and NET health care professionals
Source: Int J Cancer. 2019 Oct 25;146(5):1316–23. doi: 10.1002/ijc.32678 (PMC7004101; doi:10.1002/ijc.32678)
Supplement: Supplementary file 1 — Appendix S1: Supporting Information [file IJC-146-1316-s001.pdf]

INCA International Neuroendocrine Cancer Alliance

Unmet Needs Survey Questions – Healthcare Professionals

Q2: What is your medical expertise?

- **Oncology**
- **Pathology**
- **Radiology**
- **Nuclear medicine**
- **Endocrinology**
- **Surgery**
- **Gastroenterology**
- **Nurse**
- **Pulmonologist**
- **Other (please specify)**

Q3: Do you feel able to give sufficient information regarding NETs to patients and families?

- **Yes**
- **No, please state why**

Q4: Do you feel that you, or an appropriate member of you team, are able to spend enough discussion time with patients?

- **Needs are fully met**
- **Needs are mostly met**
- **Needs are sometimes met**
- **Needs are often not met**
- **Needs are not at all met**
- **No opinion**

Q5: Do you feel that the healthcare system in which you work supports the mental and emotional health of patients?

- **Needs are fully met**
- **Needs are mostly met**
- **Needs are sometimes met**
- **Needs are often not met**
- **Needs are not at all met**
- **No opinion**

Q6: What type of information are you able to provide and how well are needs met?

	**Needs are fully met**	**Needs are mostly met**	**Needs are sometimes met**	**Needs are often not met**	**Needs are not at all met**	**No opinion**
Information about the condition						
Information about treatment options						
Information about the doctor and/or multidisciplinary team						
Information about patient association(s) and how to contact them						
Information how to manage the condition e.g. diet, exercise, symptom control						
Information about clinical trials in NETs						
Information about research in NETs						
Information about psychological care						

Q7: Which of the following services are offered and how effective are they in meeting needs?

	**Needs are fully met**	**Needs are mostly met**	**Needs are sometimes met**	**Needs are often not met**	**Needs are not at all met**	**No opinion**
Psychological support						
Diagnosis of mental health conditions						
Treatment for diagnosed mental health conditions						
Support with continuing work and/or educations						
Palliative care						
Personal care/hygiene						

Q8: Do you utilise any of the following technologies as part of delivering care and how well do they contribute to meeting your objectives of patient care?

	Needs are fully met	Needs are mostly met	Needs are sometimes met	Needs are often not met	Needs are not at all met	I do not use it
Telephone clinics						
Web/Skype clinics						
Software or apps						
Social media						
Special equipment or devices for monitoring of their condition						

Q9: Is patient care at your hospital managed by a multidisciplinary team?

- **Yes**
- **Sometimes**
- **No**
- **No, but I am able to refer patients to a MDT at a different hospital**

Q10: How often are your patients usually in contact with a member or members of the multidisciplinary team?

- **Weekly**
- **Monthly**
- **Every 3 months**
- **Every 6 months**
- **Less regularly**

Q11: Do you feel that your level of contact with the multidisciplinary team at your hospital is appropriate?

- **Needs are fully met**
- **Needs are mostly met**
- **Needs are sometimes met**
- **Needs are often not met**
- **Needs are not at all met**

Q12: Is there a diagnostic technology that is not available to patients at your hospital currently, that you believe should be offered in appropriate cases?

- **Yes**
- **No**
- **Don't know**

Q13: Which diagnostics are not available at your hospital?

- **Gallium-68-Dotatate PET/CT scan**
- **CT**
- **MRI**
- **FDG PET**
- **MIGB**
- **Other (please specify)**

Q14: Is there a treatment option that is not available to patients at your hospital currently, that you believe should be offered in appropriate cases?

- **Yes**
- **No**
- **Don't know**

Q15: Which treatment or treatments are not available at your hospital?

- **PRRT**
- **Somatostatin analogues**
- **Chemotherapy**
- **Surgery**
- **Transplantation**
- **Interferon**
- **Liver embolization**
- **Thermal ablation**
- **Genetic testing/precision medicine**
- **Other (please specify)**

Q17: What is the furthest one of your patients ever had to travel for treatment or consultation?

- **Under 20 km/12.5 miles**
- **Up to 50 km/31 miles**
- **Up to 100 km/62 miles**
- **Up to 200 km/124 miles**
- **Up to 300 km/186 miles**
- **More than 300 km/186 miles**
- **Don't know**

Q18: Approximately how regularly do you refer your patients for treatment in another country?

- **Once a week or more**
- **Once a month**
- **A handful of patients per year**
- **Once every few years**
- **Never**
- **Don't know**

Q19: Approximately how regularly do you treat patients from another country?

- **Once a week or more**
- **Once a month**
- **A handful of patients per year**
- **Once every few years**
- **Never**
- **Don't know**

Q20: In terms of finance, to what extent does the framework within which you work allow for the appropriate delivery of care at your hospital?

	Needs are fully met	Needs are mostly met	Needs are sometimes met	Needs are often not met	Needs are not at all met	Not applicable
Diagnostic tests						
In person consultation and discussion with patients						
Treatment - surgery						
Treatment - reducing tumor progression and symptom reduction						
Follow up						
Holding multidisciplinary team meetings						
Delivery of information to patients and their families						
Participation in research						
Participation in clinical trials						
Teaching time and training for staff members						
Referral to non NET specific care e.g. dietary advice, palliative care, mental health services						

Q21: Is it made possible for you to ensure that patients have appropriate waiting times for treatment?

- **Needs are fully met**
- **Needs are mostly met**
- **Needs are sometimes met**
- **Needs are often not met**
- **Needs are not at all met**

Q22: How important a role do patient associations play within individual NET care locally, nationally or internationally?

- **Very important**
- **Somewhat important**
- **No opinion**
- **Not very important**
- **Not at all important**

Q23: How important is it that patients are involved in research design?

- **Very important**
- **Somewhat important**
- **No opinion**
- **Not very important**
- **Not at all important**

Q24: Do you feel that patients are involved enough in research design?

- **Yes**
- **No**
- **No opinion**

Q25: How important is patient involvement in specific aspects of research?

	Very important	Somewhat important	Not very important	Not at all important	No opinion
The overall strategy and direction of research funding in the field of NETs					
The design of specific research initiatives					
Interpreting the results of a research initiative to ensure published results will be understood by patients					
Setting priorities for research					
Putting NET rare cancer research on equal footing with other major forms of cancer research					
Clinical trials to improve current treatments, test new ones					

Q26: Please rank the following types of research in order of their importance to you, where 1 is most important and 7 least important

	1	2	3	4	5	6	7
Basic science to understand what causes tumors to form and grow as a pathway to discovering a cure							
Basic or translational research focused on longer survival							
Research focused on improving quality of life; controlling/managing symptoms							
Accurate, minimally invasive disease monitoring							
Earlier, more accurate diagnosis							
Clinical trials to improve current treatments, test new ones							
Development of national and international NET patient registries							

Q27: Does your hospital participate in clinical trials on neuroendocrine tumors?

- **Yes**
- **No**

Q28: How many clinical trials on neuroendocrine tumors are currently ongoing?

- **One**
- **Two**
- **Three**
- **More than three**

Q29: Has your hospital participated in a cross border clinical trial on neuroendocrine tumors?

- **Yes**
- **No**
- **Don't know**

Q30: How often have you referred late stage NET patients for inclusion in clinical trials, based upon compassionate use reasons?

- **Monthly**
- **Every 3 months**
- **Every 6 months**
- **Less regularly**
- **Never**

Q31: Do you proactively research clinical trials that are relevant to you?

- **Yes**
- **No**

Q32: How do you find out about clinical trial plans in development, in order to become involved?

- **Direct approach from clinical trial leads**
- **Printed information**
- **Medical publications and journals**
- **Online (clinicaltrials.gov etc.)**
- **Regional or international medical associations (e.g. ENETS, ESMO, NANETS)**
- **Other (please specify)**

Q33: How well are patient needs met in terms of public information that they can understand regarding ongoing or upcoming clinical trials that are relevant?

- **Needs are fully met**
- **Needs are mostly met**
- **Needs are sometimes met**
- **Needs are often not met**
- **Needs are not at all met**
- **No opinion**

Unmet Needs Survey Questions - Patient Leaders

Q2: What is your age?

- **Under 18**
- **18-29**
- **30-39**
- **40-49**
- **50-59**
- **60-69**
- **70 and over**

Q3: Are you a patient yourself, as well as leader of a patient support and/or advocacy group?

- **Yes**
- **No**

Q4: What specific type of neuroendocrine tumor has been diagnosed? (tick more than one if applicable)

- **Lung**
- **Thymic**
- **Gastric**
- **Duodenal**
- **Pancreatic**
- **Small Intestine**
- **Appendiceal**
- **Colon**
- **Rectal**
- **Ovarian**
- **Neuroendocrine tumor of unknown origin/primary**
- **Multiple Endocrine Neoplasia (MEN 1 &2)**
- **Gastrinomas**
- **Insulinomas**
- **Glucagonomas**
- **Pheocromocytoma/paraganglioma (PPGL)**
- **VIPomas**
- **Somatostatinomas**
- **Goblet Cell Carcinoma**
- **Merkel Cell Carcinoma (skin NET)**
- **Don't know**

Q5: What was your age at the time of your first NET diagnosis?

- **Under 18**
- **18-29**
- **30-39**
- **40-49**
- **50-59**
- **60-69**
- **70 and over**

Q6: When patients are first diagnosed, are they given useful information on neuroendocrine tumours?

	**Needs are fully met**	**Needs are mostly met**	**Needs are sometimes met**	**Needs are often not met**	**Needs are not at all met**
Information about the medical condition					
Information about treatment options					
Information about the doctor or multidisciplinary team					
Information about patient association(s) of relevance and their contact details					
Information how to manage the condition e.g. diet, exercise, symptom control					
Information about relevant clinical trials					
Information about NET research					
Information about psychological care					

Q7: Who gives the information?

- **Healthcare professional**
- **Patient organisation**
- **Medical funding or insurance provider**
- **Friend or family member**
- **Other (please specify)**

Q8: Do patients find for themselves or request information regarding NET e.g. information about the condition, diagnosis and treatment?

- **Yes**
- **No**

Q9: Where do they find or request information from?

- **Healthcare professional website (e.g. European Neuroendocrine Tumour Society ENETS, North American Neuroendocrine Tumor Society NANETS, a hospital's website)**
- **Healthcare professional or treating hospital - printed information**
- **Patient association website**
- **Patient association - printed information**

Q10: How well does the available information, found or requested, meet patient needs?

	**Needs are fully met**	**Needs are mostly met**	**Needs are sometimes met**	**Needs are often not met**	**Needs are not at all met**	**Not applicable**
Healthcare professional website (e.g. ENETS, NANETS, a hospital's website)						
Healthcare professional or treating hospital - printed information						
Patient association website						

Patient association - printed information						

Q11: Do patients have access to a personal home care-giver that is someone who, for example, helps with personal care, hygiene, food shopping and preparation?

- **Needs are fully met**
- **Needs are mostly met**
- **Needs are sometimes met**
- **Needs are often not met**
- **Needs are not at all met**
- **Don't know**

Q12: How much time, per week, is the personal care giver available?

- **1-10 hours**
- **11-20 hours**
- **21-30 hours**
- **More than 30 hours**
- **Don't know**

Q13: Who is the care provided by and how well are needs met?

	**Needs are fully met**	**Needs are mostly met**	**Needs are sometimes met**	**Needs are often not met**	**Needs are not at all met**	**Don't know**
A friend or family member						
A professional home caregiver who is not the patient's NET doctor						
A combination of both						

Q14: Other than specific NET treatments, how well do you feel that patients' health is supported by healthcare providers, in terms of the following needs?

	**Needs are fully met**	**Needs are mostly met**	**Needs are sometimes met**	**Needs are often not met**	**Needs are not at all met**	**Don't know**
Psychological care						
Information how to manage the condition e.g. diet, exercise, symptom control						
Diagnosis of mental health conditions						
Treatment for diagnosed mental health conditions						
Support with continuing work and/or educations						
Treatment for pain						
Personal care/hygiene						
Palliative care						

Q15: Do patients utilize any of the following technologies as part of their care, and if so how helpful are they?

	Needs are fully met	Needs are mostly met	Needs are sometimes met	Needs are often not met	Needs are not at all met	Not being used by patients
Telephone clinics						
Web/Skype clinics						
Software or apps						
Social media						
Special equipment or devices for monitoring of your condition						

Q16: How much do you agree with the following statements regarding NET patient care?

	Needs are fully met	Needs are mostly met	Needs are sometimes met	Needs are often not met	Needs are not at all met	No opinion
Patients are given all the information and explanations needed about their condition and treatment options						
Patients are able to find for themselves all of the information they need about their condition and treatment options						
Patients feel like they are truly a partner alongside the healthcare professionals (nurses, doctors, etc) in treatment and care decisions						
The healthcare financial system where I live facilitates an appropriate standard of care for patients						

Q17: In your patient support or advocacy group's region of interest, is patient care ever managed by a multidisciplinary team?

- **Yes**
- **No**
- **Don't know**

Q18: How often are patients usually in contact with a member or members of a multidisciplinary team?

- **Weekly**
- **Monthly**
- **Every 3 months**
- **Every 6 months**
- **Once per year**
- **Less regularly**

Q19: Do you feel that patients have appropriate, regular, contact with the multidisciplinary team?

- **Needs are fully met**
- **Needs are mostly met**
- **Needs are sometimes met**
- **Needs are often not met**
- **Needs are not at all met**
- **I have no opinion**

Q20: Is there a diagnostic option that is not available to patients, that they feel and/or have discussed with healthcare professionals or multidisciplinary team as possibly being appropriate?

- **Yes**
- **No**
- **Don't know**

Q21: Which diagnostic option is not available?

- **Gallium-68-Dotatate PET/CT scan**
- **Computerized tomography (CT) scan**
- **Magnetic resonance imaging (MRI) scan**
- **Fluorodeoxyglucose (FDG)-positron emission tomography (PET) scan**
- **MIBG radiopharmaceutical scan**
- **Ultrasound**
- **Other (please specify)**

Q22: Is there a treatment option that is not available to patients, that they feel and/or have discussed with the healthcare professional or multidisciplinary team as possibly being appropriate?

- **Yes**
- **No**
- **Don't know**

Q23: Which treatment or treatments are not available?

- **PRRT**
- **Somatostatin analogues**
- **Chemotherapy**
- **Surgery**
- **Transplantation**
- **Interferon**
- **Liver embolization**
- **Thermal ablation**
- **Genetic testing/precision medicine**
- **Other (please specify)**

Q24: If you know why the treatment is not available, please tick the reason(s)

- **Health insurance does not cover this treatment**
- **The state healthcare system does not make this treatment available**
- **Patients have to pay out of pocket for the treatment, and cannot afford it**
- **Patients live too far away from a centre that provides the treatment**
- **Age restrictions on the treatmaent**
- **Other (please specify)**

Q25: What is the furthest patients have to travel for treatment or consultation with a NET specialist?

- **Under 20 km/12.5 miles**
- **Up to 50 km/31 miles**
- **Up to 100 km/62 miles**
- **Up to 200 km/124 miles**
- **Up to 300 km/186 miles**
- **More than 300 km/186 miles**

Q26: Do patients ever travel to a different country for treatment?

- **Yes**
- **No**

Q27: How important a role do patient associations, either local, national or international, play in helping patients get access to appropriate NET care?

- **Very important**
- **Somewhat important**
- **No opinion**
- **Not very important**
- **Not at all important**

Q28: How important is it that patients are involved in research design?

- **Very important**
- **Somewhat important**
- **No opinion**
- **Not very important**
- **Not at all important**

Q29: Do you feel that patients are involved enough in research design?

- **Yes**
- **No**
- **No opinion**

Q30: How important is patient involvement in specific aspects of research?

	**Very important**	**Somewhat important**	**Not very important**	**Not at all important**	**No opinion**
The overall strategy and direction of research funding in the field of NETs					
The design of specific research initiatives					
Interpreting the results of a research initiative to ensure published results will be understood by patients					
Setting priorities for research					
Putting NET rare cancer research on equal footing with other major forms of cancer research					
Clinical trials to improve current treatments, test new ones					

Q31: Please rank the following types of research in order of their importance to you, where 1 is most important and 7 least important

	1	2	3	4	5	6	7
Basic science to understand what causes tumors to form and grow as a pathway to discovering a cure							
Basic or translational research focused on longer survival							
Research focused on improving quality of life; controlling/managing symptoms							
Accurate, minimally invasive disease monitoring							
Earlier, more accurate diagnosis							
Clinical trials to improve current treatments, test new ones							
Development of national and international NET patient registries							

Q32: How well does the healthcare system in your region of interest facilitate the enrolment of patients in clinical trials?

- **Needs are fully met**
- **Needs are mostly met**
- **Needs are sometimes met**
- **Needs are often not met**
- **Needs are not at all met**

Q33: Can enrolment in clinical trials made a positive contribution to appropriate patients' treatment regimes?

- **Very positive**
- **Mostly positive**
- **Somewhat positive**
- **Neither positive nor negative**
- **Somewhat negative**
- **Mostly negative**
- **Very negative**

Q34: Do you research clinical trials that are relevant?

- **Yes**
- **No**

Q35: Where do patients find out about relevant clinical trials and how well does the information available meet their needs?

	**Needs are fully met**	**Needs are mostly met**	**Needs are sometimes met**	**Needs are often not met**	**Needs are not at all met**	**Information source is not used**
Healthcare professional(s)						
Patient organisation(s)						
Printed information						
Online (clinicaltrials.gov etc)						

Unmet Needs Survey Questions - Patients and Family

Q2: Are you a patient?

- **Yes**
- **No**

Q3: Are, or were, you a family member or friend of a patient

- **Yes**
- **No**

Q4: What specific type of neuroendocrine tumor has been diagnosed? (tick more than one if applicable)

- **Lung**
- **Thymic**
- **Gastric**
- **Duodenal**
- **Pancreatic**
- **Small Intestine**
- **Appendiceal**
- **Colon**
- **Rectal**
- **Ovarian**
- **Neuroendocrine tumor of unknown origin/primary**
- **Multiple Endocrine Neoplasia (MEN 1 &2)**
- **Gastrinomas**
- **Insulinomas**
- **Glucagonomas**
- **Pheocromocytoma/paraganglioma (PPGL)**
- **VIPomas**
- **Somatostatinomas**
- **Goblet Cell Carcinoma**
- **Merkel Cell Carcinoma (skin NET)**
- **Don't know**

Q5: What is your age?

- **Under 18**
- **18-29**
- **30-39**
- **40-49**
- **50-59**
- **60-69**
- **70 and over**

Q6: What was your age at the time of your first NET diagnosis?

- **Under 18**
- **18-29**
- **30-39**
- **40-49**
- **50-59**
- **60-69**
- **70 and over**

Q7: When you were first diagnosed, were you given useful information on neuroendocrine tumours?

	**My needs were fully met**	**My needs were mostly met**	**My needs were sometimes met**	**My needs were often not met**	**My needs were not at all met**
Information about my medical condition					
Information about treatment options					
Information about your doctor or multidisciplinary team					
Information about patient association(s) relevant to you and their contact details					
Information how to manage the condition e.g. diet, exercise, symptom control					
Information about relevant clinical trials					
Information about NET research					
Information about psychological care					

Q8: Who gave you the information?

- **Healthcare professional**
- **Patient organisation**
- **Medical funding or insurance provider**
- **Friend or family member**
- **Other (please specify)**

Q9: Did you find for yourself or request information regarding NET e.g. information about your condition, diagnosis and treatment?

- **Yes**
- **No**

Q10: Where did you find or request information from?

- **Healthcare professional website (e.g. European Neuroendocrine Tumour Society ENETS, North American Neuroendocrine Tumor Society NANETS, a hospital's website)**
- **Healthcare professional or treating hospital - printed information**
- **Patient association website**
- **Patient association - printed information**

Q11: How well did the information you found or requested meet your needs?

	**My needs are fully met**	**My needs are mostly met**	**My needs are sometimes met**	**My needs are often not met**	**My needs are not at all met**	**Not applicable to me**
Healthcare professional website (e.g. ENETS, NANETS, a hospital's website)						
Healthcare professional or treating hospital - printed information						
Patient associate website						
Patient association - printed information						

Q12: Do you have a personal home care-giver that is someone who, for example, helps with personal care, hygiene, food shopping and preparation?

- **Yes**
- **No**
- **Not applicable - I don't need one**

Q13: How much time, per week, is a personal care giver available for you?

- **0 hours**
- **1-10 hours**
- **11-20 hours**
- **21-30 hours**
- **More than 30 hours**

Q14: Who is the care provided by and how well are your needs met?

	My needs are fully met	My needs are mostly met	My needs are sometimes met	My needs are often not met	My needs are not at all met	Not applicable to me
A friend or family member						
A professional home caregiver who is not the patient's NET doctor						
A combination of both						

Q15: Other than your specific NET treatments, how well do you feel that your health is supported by your healthcare providers, in terms of the following needs

	My needs are fully met	My needs are mostly met	My needs are sometimes met	My needs are often not met	My needs are not at all met	Not applicable to me
Psychological care						
Information how to manage the condition e.g. diet, exercise, symptom control						
Diagnosis of mental health conditions						
Treatment for diagnosed mental health conditions						
Support with continuing work and/or educations						
Treatment for pain						
Personal care/hygiene						
Palliative care						

Q16: Do you utilize any of the following technologies as part of your care, and if so how helpful are they to your treatment/care?

	**My needs are fully met**	**My needs are mostly met**	**My needs are sometimes met**	**My needs are often not met**	**My needs are not at all met**	**I don't use it**
Telephone clinics						
Web/Skype clinics						
Software or apps						
Social media						
Special equipment or devices for monitoring of your condition						

Q17: How much do you agree with the following statements regarding your care?

	My needs are fully met	My needs are mostly met	My needs are sometimes met	My needs are often not met	My needs are not at all met	No opinion
I am given all the information and explanations needed about my condition and my treatment options						
I am able to find for myself all of the information I need about my condition and my treatment options						
I feel like I am truly a partner alongside my healthcare professionals (nurses, doctors, etc) in treatment and care decisions						
The healthcare financial system where I live facilitates an appropriate standard of care for me						

Q18: Is your care managed by a multidisciplinary team?

- **Yes**
- **No**
- **Don't know**

Q19: How often are you in contact with a member or members of your multidisciplinary team?

- **Weekly**
- **Monthly**
- **Every 3 months**
- **Every 6 months**
- **Once per year**
- **Less regularly**

Q20: Do you feel that you have appropriate, regular, contact with the multidisciplinary team?

- **My needs are fully met**
- **My needs are mostly met**
- **My needs are sometimes met**
- **My needs are often not met**
- **My needs are not at all met**
- **I have no opinion**

Q21: Is there a diagnostic option that is not available to you, that you feel and/or have discussed with your healthcare professional or multidisciplinary team as possibly being appropriate?

- **Yes**
- **No**
- **Don't know**

Q22: Which diagnostic option is not available to you?

- **Gallium-68-Dotatate PET/CT scan**
- **Computerized tomography (CT) scan**
- **Magnetic resonance imaging (MRI) scan**
- **Fluorodeoxyglucose (FDG)-positron emission tomography (PET) scan**
- **MIBG radiopharmaceutical scan**
- **Ultrasound**
- **Other (please specify)**

Q23: Is there a treatment option that is not available to you, that you feel and/or have discussed with your healthcare professional or multidisciplinary team as possibly being appropriate?

- **Yes**
- **No**
- **Don't know**

Q24: Which treatment or treatments are not available to you?

- **PRRT**
- **Somatostatin analogues**
- **Chemotherapy**
- **Surgery**
- **Transplantation**
- **Interferon**
- **Liver embolization**
- **Thermal ablation**
- **Genetic testing/precision medicine**
- **Other (please specify)**

Q25: If you know why the treatment is not available to you, please tick the reason(s)

- **My health insurance does not cover this treatment**
- **The state healthcare system does not make this treatment available**
- **I have to pay out of pocket for the treatment, and I cannot afford it**
- **I live too far away from a centre that provides the treatment**
- **My physician will not refer me for the treatment**
- **I am considered an inappropriate age for this treatment**
- **Other (please specify)**

Q26: What is the furthest you ever had to travel for treatment or consultation with a NET specialist?

- **Under 20 km/12.5 miles**
- **Up to 50 km/31 miles**
- **Up to 100 km/62 miles**
- **Up to 200 km/124 miles**
- **Up to 300 km/186 miles**
- **More than 300 km/186 miles**

Q27: Did you ever travel to a different country for treatment?

- **Yes**
- **No**

Q28: How important a role do patient associations, either local, national or international, play in helping you get access to appropriate NET care?

- **Very important**
- **Somewhat important**
- **No opinion**
- **Not very important**
- **Not at all important**

Q29: How important is it that patients are involved in research design?

- **Very important**
- **Somewhat important**
- **No opinion**
- **Not very important**
- **Not at all important**

Q30: Do you feel that patients are involved enough in research design?

- **Yes**
- **No**
- **No opinion**

Q31: How important is patient involvement in specific aspects of research?

	Very important	Somewhat important	Not very important	Not at all important	No opinion
The overall strategy and direction of research funding in the field of NETs					
The design of specific research initiatives					
Interpreting the results of a research initiative to ensure published results will be understood by patients					
Setting priorities for research					
Putting NET rare cancer research on equal footing with other major forms of cancer research					
Clinical trials to improve current treatments, test new ones					

Q32: Please rank the following types of research in order of their importance to you, where 1 is most important and 7 least important

	1	2	3	4	5	6	7
Basic science to understand what causes tumors to form and grow as a pathway to discovering a cure							
Basic or translational research focused on longer survival							
Research focused on improving quality of life; controlling/managing symptoms							
Accurate, minimally invasive disease monitoring							
Earlier, more accurate diagnosis							
Clinical trials to improve current treatments, test new ones							
Development of national and international NET patient registries							

Q33: Have you ever been enrolled in a neuroendocrine tumor clinical trial?

- **Yes**
- **No**

Q34: How many neuroendocrine tumor clinical trials have you been enrolled in?

- **One**
- **Two**
- **Three**
- **More than three**

Q35: Has enrolment in clinical trials made a positive contribution to your treatment regime?

- **Very positive**
- **Mostly positive**
- **Somewhat positive**
- **Neither positive nor negative**
- **Somewhat negative**
- **Mostly negative**
- **Very negative**

Q36: Do you research clinical trials that are relevant to you?

- **Yes**
- **No**

Q37: Where did you find out about relevant clinical trials and how well did the information meet your needs?

	**My needs are fully met**	**My needs are mostly met**	**My needs are sometimes met**	**My needs are often not met**	**My needs are not at all met**	**I don't use this source of information**
Healthcare professional(s)						
Patient organisation(s)						
Printed information						
Online (clinicaltrials.gov etc.)						
