# Supplementary figures and images for: Unmet needs in the international neuroendocrine tumor (NET) community: Assessment of major gaps from the perspective of patients, patient advocates and NET health care professionals
Source: Int J Cancer. 2019 Oct 25;146(5):1316–23. doi: 10.1002/ijc.32678 (PMC7004101; doi:10.1002/ijc.32678)

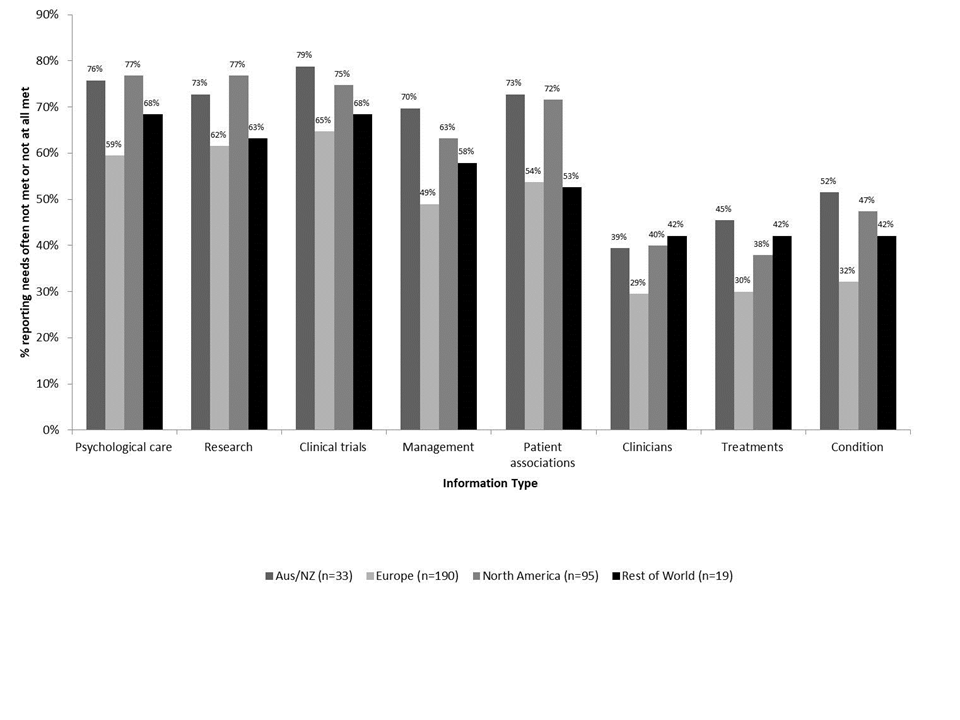

Supplement: Supplementary file 2 — Supplementary Figure 1 Patient unmet informational needs at diagnosis by region (online only) Aus: Australia; NZ: New Zealand; Europe: Austria, Belgium, Bulgaria, Denmark, Finland, France, Germany, Ireland, Italy, The Netherlands, Norway, Poland, Portugal, Sweden, Switzerland, Spain, United Kingdom; North America: Canada; United States of America; Rest of World: India, Japan, Nepal, Singapore, United Arab Emirates. [file IJC-146-1316-s002.tif]
