# Supplementary material for: Unmet needs in the international neuroendocrine tumor (NET) community: Assessment of major gaps from the perspective of patients, patient advocates and NET health care professionals
Source: Int J Cancer. 2019 Oct 25;146(5):1316–23. doi: 10.1002/ijc.32678 (PMC7004101; doi:10.1002/ijc.32678)
Supplement: Supplementary file 3 — Supplementary Table 1 Geographic breakdown of participants (online only) HCP: healthcare professional; UAE: United Arab Emirates; UK: United Kingdom; USA: United States of America. [file IJC-146-1316-s003.docx]

**Supplementary Table 1 Geographic breakdown of participants**

| **Countries** | **Patient/family**  **(*N*=338)** | **Advocate**  **(*N*=35)** | **HCP**  **(*N*=70)** |
| --- | --- | --- | --- |
| Australia | 20 | 2 | 13 |
| Austria | 3 | 0 | 0 |
| Belgium | 14 | 4 | 7 |
| Bulgaria | 2 | 2 | 2 |
| Canada | 18 | 1 | 4 |
| Denmark | 3 | 0 | 0 |
| Finland | 1 | 0 | 0 |
| France | 9 | 2 | 9 |
| Germany | 53 | 2 | 6 |
| India | 9 | 1 | 4 |
| Ireland | 15 | 2 | 2 |
| Italy | 0 | 2 | 1 |
| Japan | 7 | 0 | 12 |
| Nepal | 1 | 0 | 0 |
| Netherlands | 1 | 0 | 0 |
| Norway | 6 | 1 | 0 |
| New Zealand | 13 | 3 | 6 |
| Poland | 1 | 0 | 0 |
| Portugal | 1 | 0 | 0 |
| Sweden | 1 | 0 | 0 |
| Switzerland | 3 | 0 | 0 |
| Singapore | 2 | 1 | 0 |
| Spain | 0 | 1 | 0 |
| UAE | 1 | 0 | 0 |
| UK | 77 | 6 | 1 |
| USA | 78 | 5 | 3 |
