# Supplementary material for: Unmet needs in the international neuroendocrine tumor (NET) community: Assessment of major gaps from the perspective of patients, patient advocates and NET health care professionals
Source: Int J Cancer. 2019 Oct 25;146(5):1316–23. doi: 10.1002/ijc.32678 (PMC7004101; doi:10.1002/ijc.32678)
Supplement: Supplementary file 4 — Supplementary Table 2 Most common diagnostics and treatments not available (online only) CT: computerized tomography; FDG: fluorodeoxyglucose; HCP: healthcare professional; MIGB: meta‐iodobenzylguanidine radiopharmaceutical scan; PET: positron‐emission tomography; PRRT: peptide receptor radionuclide therapy. The total number of respondents in each participant group differed slightly for each question because participants were allowed to skip questions. Percentages shown are calculated based on actual numbers. [file IJC-146-1316-s004.doc]

**Supplementary Table 2 Most common diagnostics and treatments not available**

| **Diagnostics** | | | |
| --- | --- | --- | --- |
|  | **Patient/family**  **(*N*=98)** | **Advocate**  **(*N*=20)** | **HCP**  **(*N*=44)** |
| Gallium-68-Dotatate PET/CT scan, *N*, *(%)* | 71 (72) | 17 (85) | 38 (86) |
| FDG PET, *N,* (%) | 23 (23) | 2 (10) | 16 (36) |
| MIGB, *N,* (%) | 18 (18) | 1 (5) | 10 (23) |
| **Treatments** | | | |
|  | **Patient/family**  **(*N*=107)** | **Advocate**  **(*N*=21)** | **HCP**  **(*N*=56)** |
| PRRT, *N*, (%) | 45 (42) | 20 (95) | 43 (77) |
| Genetic testing/precision medicine, *N*, (%) | 27 (25) | 7 (33) | 16 (29) |
| Surgery, *N*, (%) | 20 (19) | 0 (0) | 9 (16) |
| Transplantation, *N*, (%) | 15 (14) | 2 (10) | 25 (45) |
