# Supplementary material for: Unmet needs in the international neuroendocrine tumor (NET) community: Assessment of major gaps from the perspective of patients, patient advocates and NET health care professionals
Source: Int J Cancer. 2019 Oct 25;146(5):1316–23. doi: 10.1002/ijc.32678 (PMC7004101; doi:10.1002/ijc.32678)
Supplement: Supplementary file 5 — Supplementary Table 3 Most common diagnostics and treatments not available by region (online only) CT: computerized tomography; FDG: fluorodeoxyglucose; MIGB: meta‐iodobenzylguanidine radiopharmaceutical scan; PET: positron‐emission tomography; PRRT: peptide receptor radionuclide therapy; Aus: Australia; NZ: New Zealand; Europe: Austria, Belgium, Bulgaria, Denmark, Finland, France, Germany, Ireland, Italy, Norway, Poland, Portugal, Spain, Sweden, Switzerland, The Netherlands, United Kingdom; North America: Canada, United States of America; Rest of World: India, Japan, Nepal, Singapore, the United Arab Emirates. Values shown are calculated based on actual numbers (respondents were allowed to skip questions), and is combined for all participants (patient/advocate/healthcare professional) within each region. [file IJC-146-1316-s005.doc]

**Supplementary Table 3 Most common diagnostics and treatments not available by region**

| **Diagnostics** | | | | |
| --- | --- | --- | --- | --- |
|  | **Aus/NZ**  **(*N*=30)** | **Europe**  **(*N*=52)** | **North America**  **(*N*=52)** | **Rest of World**  **(*N*=28)** |
| Gallium-68-Dotatate PET/CT scan, *N*, *(%)* | 22 (73) | 42 (81) | 47 (90) | 14 (50) |
| FDG PET, *N,* (%) | 10 (33) | 14 (27) | 11 (21) | 6 (21) |
| MIGB, *N,* (%) | 9 (30) | 10 (19) | 6 (12) | 4 (14) |
| **Treatments** | | | | |
|  | **Aus/NZ**  **(*N*=40)** | **Europe**  **(*N*=64)** | **North America**  **(*N*=52)** | **Rest of World**  **(*N*=28)** |
| PRRT, *N*, (%) | 21 (53) | 37 (58) | 35 (67) | 13 (46) |
| Genetic testing/precision medicine, *N*, (%) | 2 (5) | 16 (25) | 10 (19) | 5 (18) |
| Surgery, *N*, (%) | 5 (13) | 13 (20) | 7 (13) | 4 (14) |
| Transplantation, *N*, (%) | 7 (18) | 18 (28) | 8 (15) | 7 (25) |
